# A population-based recurrence risk management study of patients with pT1 node-negative HER2+ breast cancer: a National Clinical Database study

**DOI:** 10.1007/s10549-019-05413-7

**Published:** 2019-08-26

**Authors:** Makoto Kubo, Masaaki Kawai, Hiraku Kumamaru, Hiroaki Miyata, Kenji Tamura, Masayuki Yoshida, Etsuyo Ogo, Masayuki Nagahashi, Sota Asaga, Yasuyuki Kojima, Takayuki Kadoya, Kenjiro Aogi, Naoki Niikura, Minoru Miyashita, Kotaro Iijima, Naoki Hayashi, Yutaka Yamamoto, Shigeru Imoto, Hiromitsu Jinno

**Affiliations:** 1 Department of Surgery and Oncology, Graduate School of Medical Sciences, Kyushu University, 3-1-1 Maidashi Higashi-ku, Fukuoka, 812-8582 Japan; 2 Department of Breast Oncology, Miyagi Cancer Center Hospital, 47-1 Nodayama, Medeshima-Shiode, Natori, Miyagi 981-1293 Japan; 3 Department of Healthcare Quality Assessment, University of Tokyo, 7-3-1 Hongo, Bunkyo-ku, Tokyo, 113-8655 Japan; 4 Department of Breast and Medical Oncology, National Cancer Center Hospital, 5-1-1 Tsukiji, Chuo-ku, Tokyo, 104-0045 Japan; 5 Department of Diagnostic Pathology, National Cancer Center Hospital, 5-1-1 Tsukiji, Chuo-ku, Tokyo, 104-0045 Japan; 6 Department of Radiology, Kurume University School of Medicine, 67 Asahi-Machi, Kurume, Fukuoka, 830-0011 Japan; 7 Division of Digestive and General Surgery, Niigata University Graduate School of Medical and Dental Sciences, 1-757 Asahimachi-dori, Chuo-ku, Niigata, 951-8510 Japan; 8 Department of Breast Surgery, Kyorin University School of Medicine, 6-20-2 Shinkawa, Mitaka, Tokyo, 181-8611 Japan; 9 Division of Breast and Endocrine Surgery, Department of Surgery, St. Marianna University School of Medicine, 2-16-1 Sugao, Miyamae-ku, Kawasaki, 216-8511 Japan; 10 Department of Surgical Oncology, Research Institute for Radiation Biology and Medicine, Hiroshima University, 1-2-3 Kasumi, Minami-ku, Hiroshima, 734-0037 Japan; 11 Department of Breast Oncology, National Hospital Organization Shikoku Cancer Center, Kou 160, Minamiumemotomachi, Matsuyama, Ehime 791-0280 Japan; 12 Department of Breast and Endocrine Surgery, Tokai University School of Medicine, 143 Shimokasuya, Isehara, Kanagawa 259-1193 Japan; 13 Department of Breast and Endocrine Surgical Oncology, Tohoku University School of Medicine, Seiryo-machi, Aoba-ku, Sendai, 980-8574 Japan; 14 Department of Breast Oncology, Juntendo University, 3-1-3 Hongo, Bunkyo-ku, Tokyo, 113-8431 Japan; 15 Department of Breast Surgical Oncology, St. Luke’s International Hospital, 9-1 Akashicho, Chuo-ku, Tokyo, 104-8560 Japan; 16 Department of Molecular-Targeting Therapy for Breast Cancer, Kumamoto University, 1-1-1 Honjo, Chuo-ku, Kumamoto, 860-8556 Japan; 17 Department of Breast Surgery, Kyorin University Hospital, 6-20-2 Shinkawa, Mitaka, Tokyo, 181-8611 Japan; 18 Department of Surgery, Teikyo University School of Medicine, 2-11-1 Kaga, Itabashi-ku, Tokyo, 173-8606 Japan

**Keywords:** Breast cancer, Stage I, Human epidermal growth factor receptor 2, Chemotherapy, Trastuzumab

## Abstract

**Purpose:**

Recurrence risk management of patients with small (≤ 2 cm), node-negative, human epidermal growth factor receptor 2 (HER2)-positive breast cancer remains challenging. We studied the effects of adjuvant chemotherapy and/or trastuzumab and survival outcomes among these patients, using data from the population-based Japanese National Clinical Database (NCD).

**Methods:**

We identified a cohort of 2736 breast cancer patients with HER2+ pT1N0 disease: 489 pT1a, 642 pT1b, and 1623 pT1c. The median observation period was 76 months, and the 5-year follow-up rate was 48.2%. The number of events was 212 for disease-free survival (DFS), 40 for breast cancer-specific survival, and 84 for overall survival (OS).

**Results:**

There were 24.5% of pT1a, 51.9% of pT1b, and 63.3% of pT1c patients who were treated systemically after surgery. OS in pT1b (logrank test; *p* = 0.03) and DFS in pT1c (logrank test; *p* < 0.001) were significantly improved in treated compared with untreated patients. In the Cox proportional hazards model, treated patients had significantly longer OS than untreated patients in pT1b (hazard ratio (HR) 0.20) and pT1c (HR 0.54) groups. Estrogen receptor-negative tumors was also a significant predictor of survival in pT1c (HR 2.01) but not pT1ab patients. Furthermore, HR was greater in patients aged ≤ 35 years (3.18) compared to that in patients aged 50–69 years in the pT1b group.

**Conclusions:**

NCD data revealed that systemic treatment improved OS in pT1bc but not in pT1a node-negative HER2+ breast cancer patients. Future observational research using big-sized data is expected to play an important role in optimizing treatment for patients with early-stage breast cancer.

**Electronic supplementary material:**

The online version of this article (10.1007/s10549-019-05413-7) contains supplementary material, which is available to authorized users.

## Introduction

The incidence of stage I breast cancer has increased gradually to nearly half of all primary breast cancer cases because of detection of nonpalpable breast cancer using screening mammography in Japan as well as in Europe and the United States [1–5]. According to breast cancer registry, there was a marked increase from 37.3 to 51.6% in the proportion of stage I breast cancer patients during the year 2004 to 2014 [1]. Although it is well known that patients with T1ab node-negative tumors (1 cm or less) generally have a favorable prognosis, outcomes for those patients may depend largely on tumor subtypes [6, 7].

Human epidermal growth factor receptor 2 (HER2) overexpression is an independent, poor prognostic factor, and a positive predictive biomarker in response to HER2-targeted therapy [8]. HER2-overexpressing or HER2-amplified breast cancer accounts for 20–30% of invasive breast carcinomas [8], and without treatment, has the worst prognosis among subtypes [7]. Various guidelines support the use of trastuzumab-based chemotherapy as a standard option in tumors larger than 1 cm, and suggest its administration in T1b tumors because some studies have reported that HER2 overexpression was an independent, poor prognostic factor even for patients with pT1ab node-negative HER2+ breast cancer [6, 9].

Trastuzumab (Herceptin®, Roche) has been available as adjuvant systemic therapy in Japan since February 2008. Several randomized clinical trials [10–16] have shown an improved outcome among Trastuzumab users in pStage II–III disease. However, since patients with pStage I disease were mostly excluded from these trials, the evidence for benefit of chemotherapy and/or trastuzumab in those patients is limited. The aim of our study was to assess the recurrence and survival of pT1abN0 HER2+ breast cancer patients across different types of treatments and hormone receptor types, using data from the nationwide breast cancer registry, to provide information on possible treatment options to the healthcare providers.

## Patients and methods

### Data collection

The Breast Cancer Registry (BCR) run on the National Clinical Database (NCD) contains records of more than 560,000 patients with breast cancer from more than 1400 hospitals, as of 2015. Affiliated institutions provide data on newly diagnosed primary breast cancer patients through a web-based system to the BCR-NCD, covering more than 50 demographic and clinicopathologic characteristics. The initial follow-up is requested for 5-year prognosis since first treatment (preoperative therapy or surgery). The BCR-NCD was originally maintained by the Registration Committee of the Japanese Breast Cancer Society (JBCS) and was supported by the Public Health Research Foundation (Tokyo, Japan). It is currently managed by NCD, which is a platform for various nationwide registries in Japan. TNM classification is now registered according to the 7th edition of the Unio Internationalis Contra Cancrum staging system [17], and histological classification was registered according to the General Rules for Clinical and Pathological Recording of Breast Cancer [18], which was further transferred to the Classification of Tumors of the Breast and Female Genital Organs [19].

### Study patients

**Fig. 1:**
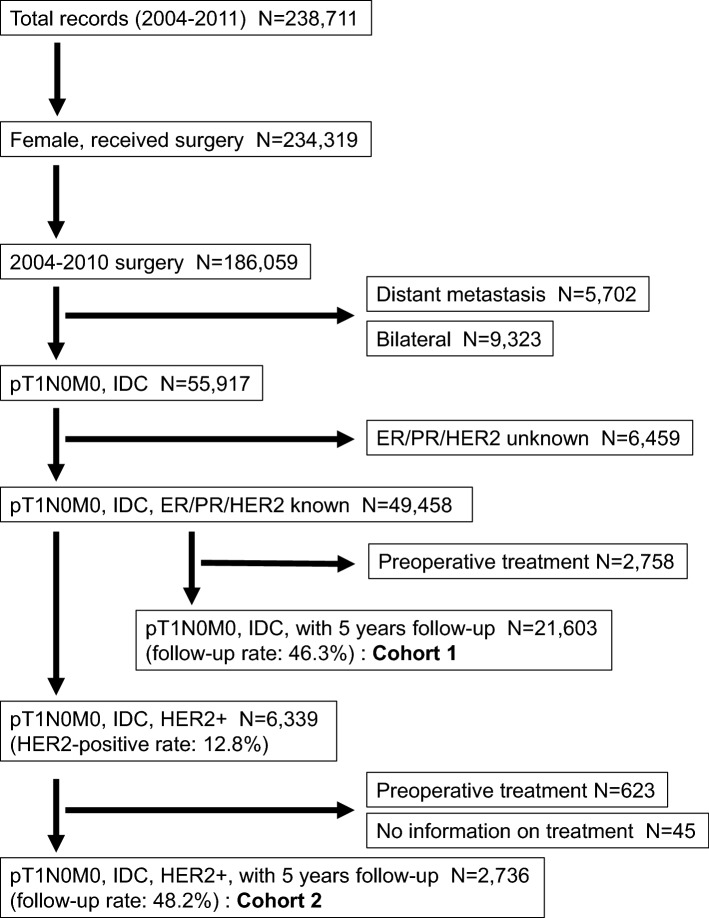
Study flow chart

For our study, among 238,711 breast cancer patients registered between 2004 and 2011, we selected 186,059 female patients who underwent surgery (Fig. 1). Patients with bilateral tumors, those who received preoperative systemic therapy, and those with distant metastases were excluded, resulting in 55,917 patients. HER2 overexpression was defined as immunohistochemistry (IHC) 3 + and/or a positive fluorescent in situ hybridization test according to the manufacturer’s criteria [20]. Hormone receptor (estrogen receptor (ER) or progesterone receptor (PR)) expression was considered positive if at least 1% of nuclei in tumor cells were stained using IHC for ER or PR. Tumor subtypes were categorized on the basis of IHC as follows: luminal A (ER+, PR ≥ 20%, and HER2−); luminal B (ER+, PR < 20%, and HER2−) [21–23]; luminal-HER2 (ER+ and/or PR+/HER2+); HER2 (ER− and PR−/HER2+); and triple negative (ER−, PR−, and HER2−). Of the 49,458 patients with hormone receptor and HER2 expression-known pT1 tumors registered in the NCD, 21,603 patients (46.3%) with 5-year follow-up data were taken up for the study and analyzed for survivals based on different subtypes (Cohort 1).

### Exposure and patient cohort

Of the 6339 patients with pT1 HER2+ tumors, 623 tumors were removed because of receiving preoperative treatment, and an additional 45 cases were removed because of a lack of treatment information, leaving 2736 patients (48.2%) with 5-year follow-up data, who were analyzed in our study (Cohort 2). Of the patients, we compared 1472 treated patients by systemic therapies (chemotherapy with or without trastuzumab) to 1265 untreated patients.

### Statistical analysis

Pearson’s Chi squared test was used to determine differences among the three patient groups, all with T1/HER2+ status. Wilcoxon’s test were used for intergroup comparisons of continuous variables.

Survival curves were constructed using the Kaplan–Meier method with and without stratification on known prognostic factors and were compared using logrank test. Multivariable survival analyses for disease-free survival (DFS), breast cancer-specific survival (BCSS), and overall survival (OS) were performed using Cox proportional hazards modeling, to estimate the hazard of systemically treated patients relative to untreated patients by pT1a-c group. We considered the following variables as potential confounders in the Cox model: age, surgery, ER and PR statuses, postoperative systemic therapy, and pathological type. Patients with any missing or unknown data were excluded from these analyses. DFS was defined as the time interval between the date of surgery and the time of local or distant recurrence. BCSS and OS were defined as the time interval between the date of surgery and the date of breast cancer-related death or death from any cause, respectively. All tests were two-sided and *a p* value < 0.05 was considered statistically significant. All analyses were carried out using SAS version 9.4 (SAS Institute, Cary, NC).

## Results

We identified a cohort of 2736 patients with HER2+ pT1N0M0 disease (Fig. 1). Patient characteristics are shown in Table 1. The median follow-up period was 76 months, and the 5-year follow-up rate was 48.2%. The number of events was 212 for DFS, 40 for BCSS, and 84 for OS.

**Table 1:** Clinicopathologic characteristics of pT1N0 HER2-positive breast cancer (cohort 2)

	pT1a		pT1b		pT1c	
	No treatment	Treatment	No treatment	Treatment	No treatment	Treatment
*N*	369	120	300	324	595	1028
Age at diagnosis						
< 35	10 (2.7%)	7 (5.8%)	9 (3.0%)	14 (4.3%)	15 (2.5%)	39 (3.8%)
35–49	79 (21.4%)	44 (36.7%)	69 (23.0%)	89 (27.5%)	124 (20.8%)	256 (24.9%)
50–69	240 (65.0%)	68 (56.7%)	172 (57.3%)	198 (61.1%)	296 (49.8%)	646 (62.8%)
≥ 70	40 (10.8%)	1 (0.8%)	50 (16.7%)	23 (7.1%)	160 (26.9%)	87 (8.5%)
Operation						
BCS	165 (44.7%)	45 (37.5%)	204 (68.0%)	185 (57.1%)	395 (66.4%)	669 (65.1%)
Mastectomy	204 (55.3%)	74 (61.7%)	94 (31.3%)	137 (42.3%)	197 (33.1%)	357 (34.7%)
Other	0	1 (0.8%)	2 (0.7%)	2 (0.6%)	3 (0.5%)	2 (0.2%)
ER						
Positive	149 (40.4%)	29 (24.2%)	212 (70.7%)	141 (43.5%)	447 (75.1%)	516 (50.2%)
Negative	220 (59.6%)	91 (75.8%)	88 (29.3%)	183 (56.5%)	148 (24.9%)	512 (49.8%)
PR						
Positive	87 (23.6%)	17 (14.2%)	143 (47.7%)	89 (27.5%)	324 (54.5%)	339 (33.0%)
Negative	282 (76.4%)	103 (85.8%)	157 (52.3%)	235 (72.5%)	271 (45.6%)	689 (67.0%)
Treatment						
Chemotherapy alone		58 (11.9%)		136 (21.8%)		384 (23.7%)
Trastuzumab alone		23 (4.7%)		45 (7.2%)		83 (5.1%)
Both*		39 (8.0%)		143 (22.9%)		561 (34.6%)
No treatment	369 (75.5%)		300 (48.1%)		595 (36.7%)	
Year of operation						
2004–2005	56 (15.2%)	25 (20.8%)	89 (29.7%)	52 (16.1%)	181 (30.4%)	158 (15.4%)
2006–2007	94 (25.5%)	29 (24.2%)	67 (22.3%)	75 (23.2%)	153 (25.7%)	203 (19.8%)
2008–2010	219 (59.4%)	66 (55.0%)	144 (48.0%)	197 (60.8%)	261 (43.9%)	667 (64.8%)
Pathological type						
Invasive ductal	347 (94.0%)	113 (94.2%)	275 (91.7%)	304 (93.8%)	553 (92.9%)	970 (94.4%)
Invasive lobular	13 (3.5%)	3 (2.5%)	2 (0.7%)	3 (0.9%)	7 (1.2%)	8 (0.8%)
Other	9 (2.4%)	4 (3.3%)	23 (7.7%)	17 (5.2%)	35 (5.9%)	50 (4.8%)

*BCS* breast-conserving surgery, *ER* estrogen receptor, *PR* progesterone receptor, both* chemotherapy + trastuzumab

In general, patients with T1c tumors had a poorer OS compared to those with T1a or T1b tumors. Among patients with HER2+ tumors, those with T1b or T1c tumors had a poorer OS compared to those with T1a tumors (Supplementary Fig. 1). Moreover, patients with luminal A and luminal-HER2 T1c tumors did not have poorer OS, whereas patients with luminal B and triple negative T1c tumors had significantly poorer OS compared to those with T1a or T1b tumors (Supplementary Fig. 2). Patients with HER2-enriched T1c and also T1b tumors had a poorer OS compared to those with T1a tumors (*p* = 0.04).

**Fig. 2:**
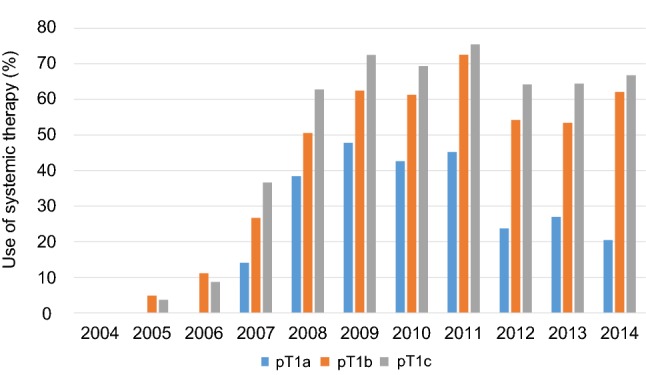
Use of systemic therapy for HER2 + breast cancer in Japan between 2004 and 2014

Prognostic information was available for 489 T1a (17.9%), 624 T1b (22.8%), and 1623 T1c patients (59.3%) (Table 1). There were 24.5% of T1a, 51.9% of T1b, and 63.3% of T1c patients who received treatment, chemotherapy, and/or trastuzumab. HER2+ patients aged < 50, those who were premenopausal, those who received breast-conserving surgery, and those who were ER−/PR− had a high probability of receiving systemic treatment, while patients with ER+ T1b tumors were less likely to be treated. Those with ER− compared with ER+ tumors tended to be treated with chemotherapy and/or trastuzumab to a greater extent (Supplementary Fig. 3A). The treatment group in the late phase (2008–2010) compared with the early phase (2004–2007) decreased by 6.8%, resulting in 23.2% for T1a; and increased by 12.9%, resulting in 57.8% for T1b; and increased by 20.0%, resulting in 71.9% for T1c (Table 1). In 2008, the use of trastuzumab was approved in Japan. Thereafter, the administration rates of chemotherapy and/or trastuzumab stabilized at 50–70% for T1b and T1c, and at 20–30% for T1a patients, in accordance with the guidelines for the late phase (Fig. 2).

**Fig. 3:**
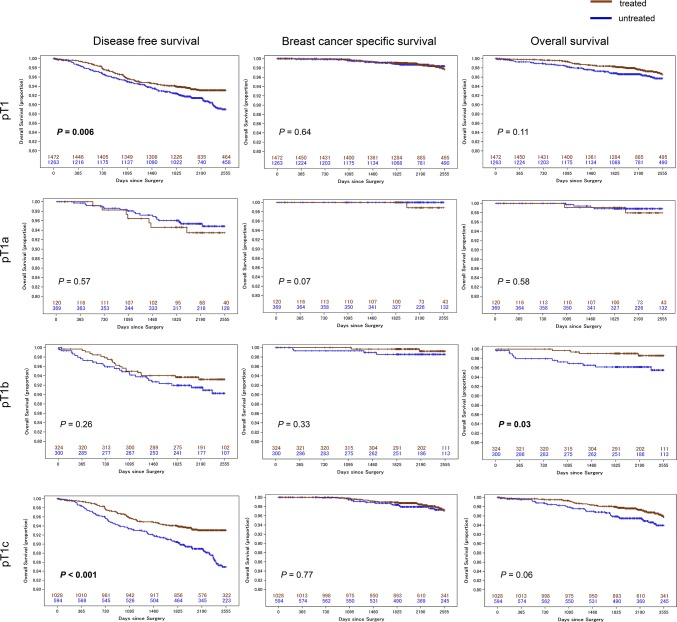
Survival curves according to systemic therapy status

There was no difference in DFS for treated versus untreated patients in either the T1a or T1b groups; however, in the T1c group, treated patients showed significantly better DFS compared with untreated patients (logrank test; *p *< 0.001) (Fig. 3).

In the Cox proportional hazards model with the background factors adjusted, the treated group had significantly longer survival than the untreated group (T1b: Hazard ratio (HR) 0.20; 95% Confidence interval (CI) 0.06–0.67 and T1c: HR 0.54; 95% CI 0.31–0.94) (Table 2). In the multivariate regression model for DFS and OS, the prognosis of treated patients was significantly better compared with untreated patients in the T1c group (DFS: HR 0.47; 95% CI 0.32–0.68 and OS: HR 0.54, 95% CI 0.31–0.94). Furthermore, treated patients had significantly better survival compared with untreated patients in the T1b group (BCSS: HR 0.17; 95% CI 0.03–0.95 and OS: HR 0.20; 95% CI 0.06–0.67). Patients with ER− tumors had significantly poorer DFS (HR 2.01, 95% CI 1.27–3.16), BCSS (HR 3.93; 95% CI 1.41–11.0), and OS (HR 2.58; 95% CI 1.27–5.24) compared with patients with ER+ tumors in the T1c group. Furthermore, in the T1b group, the hazard of patients aged 35 years or younger was significantly higher compared to the patients aged 50–69 years (HR 3.18 (95% CI 1.08–9.34)) (Table 2).

**Table 2:** Multivariate analysis of 5-year survival

Variable	DFS				BCSS				OS			
	HR	95% CI		*p* value	HR	95% CI		*p* value	HR	95% CI		*p* value
pT1												
With treatment	0.677	0.508	0.901	**0.0076**	0.906	0.471	1.742	0.088	0.695	0.442	1.094	0.1163
Age at diagnosis												
< 35	2.35	1.33	4.151	**0.0033**	3.834	1.099	13.369	4.4465	2.039	0.719	5.787	0.1806
35–49	0.988	0.686	1.421	0.9463	1.625	0.737	3.583	1.4479	0.827	0.428	1.599	0.5724
50–69	(Ref)				(Ref)				(Ref)			
≥ 70	1.844	1.287	2.643	**0.0009**	2.089	0.904	4.829	2.969	2.84	1.724	4.678	**< 0.0001**
ER												
Negative	1.561	1.083	2.248	**0.0168**	4.24	1.531	11.742	7.7255	2.036	1.12	3.702	**0.0197**
PR												
Negative	0.958	0.644	1.424	0.8304	0.966	0.312	2.993	**0.0036**	1.12	0.562	2.23	0.7477
pT1a												
With treatment	1.286	0.509	3.249	0.5945	1.575	0.272	9.109	0.9992	1.575	0.272	9.109	0.6119
Age at diagnosis												
< 35	1.909	0.386	9.447	0.4279	0	0	0	1	0	0	0	0.9968
35–49	1.221	0.481	3.096	0.6743	0.475	0.05	4.517	0.9989	0.475	0.05	4.517	0.5171
50–69	(Ref)				(Ref)				(Ref)			
≥ 70	0.775	0.098	6.112	0.809	0	0	0	1	0	0	0	0.9959
ER												
Negative	1.406	0.399	4.947	0.5959	0.817	0.099	6.731	0.999	0.817	0.099	6.731	0.8511
PR												
Negative	0.46	0.118	1.786	0.2615	0.868	0.056	13.51	0.9996	0.868	0.056	13.51	0.9193
pT1b												
With treatment	0.538	0.288	1.007	0.0526	0.169	0.03	0.947	**0.0432**	0.202	0.061	0.666	**0.0086**
Age at diagnosis												
< 35	3.177	1.081	9.335	**0.0355**	0	0	0	0.9986	2.691	0.335	21.613	0.3516
35–49	1.152	0.523	2.54	0.7252	1.723	0.177	16.812	0.6396	0.431	0.052	3.552	0.434
50–69	(Ref)				(Ref)				(Ref)			
≥ 70	1.476	0.658	3.31	0.3452	2.714	0.438	16.802	0.2831	2.1	0.69	6.396	0.1914
ER												
Negative	1.523	0.734	3.157	0.2583	0	0	0	0.996	2.604	0.728	9.314	0.141
PR												
Negative	2.249	0.936	5.403	0.0699	0	0	0	0.997	2.753	0.461	16.433	0.2666
pT1c												
With treatment	0.468	0.323	0.68	**< 0.0001**	0.572	0.265	1.237	0.1558	0.541	0.31	0.944	**0.0305**
Age at diagnosis												
< 35	2.174	1.031	4.584	**0.0414**	4.669	1.295	16.84	**0.0186**	2.229	0.663	7.491	0.1952
35–49	0.911	0.573	1.446	0.6913	1.912	0.811	4.506	0.1384	1.03	0.49	2.165	0.9379
50–69	(Ref)				(Ref)				(Ref)			
≥ 70	1.626	1.061	2.489	**0.0255**	1.394	0.523	3.717	0.5067	2.487	1.371	4.51	**0.0027**
ER												
Negative	2.005	1.273	3.158	**0.0027**	3.933	1.41	10.969	**0.0089**	2.578	1.269	5.237	**0.0088**
PR												
Negative	0.912	0.564	1.475	0.7065	1.194	0.384	3.716	0.7591	1.078	0.49	2.372	0.8511

*BCSS* breast cancer-specific survival, *CI* confidence interval, *DFS* disease-free survival, *ER* estrogen receptor, *HR* hazard ratio, *OS* overall survival, *PR* progesterone receptor: treatment, chemotherapy and/or trastuzumab, *ref* reference

## Discussion

In our study using retrospectively collected data from the Japanese BCR held within the NCD, we identified 2736 patients with early-stage pT1N0 HER2+ breast cancer and demonstrated significantly improved survival benefits (DFS: HR 0.45; 95% CI 0.32–0.68 and OS: HR 0.54; 95% CI 0.31–0.94) in those who received systemic treatment in the pT1c group (Table 2). In addition, systemic treatment improved outcomes in patients with pT1b disease (BCSS: HR 0.17; 95% CI 0.03–0.95 and OS: HR 0.20; 95% CI 0.06–0.67). Therefore, our study contributes to our understanding of whether to administer chemotherapy and/or trastuzumab to an increasing population of patients with early-stage HER2+ tumors. Such patients have generally not been included in randomized trials of adjuvant systemic therapy, and presently, there are no randomized trials that can provide level I evidence. Therefore, our observational registry study likely represents the best available and appropriate evidence on the management of benefit and harm in patients with pT1N0 HER2+ breast cancer.

Our data showed that treatment of pT1a tumors was not associated with an improved prognosis; therefore, caution should be used when deciding whether to treat patients with pT1a tumors. Whether all patients with early-stage HER2+ tumors require both adjuvant chemotherapy and 1 year of trastuzumab is still unclear. Guidelines from the National Comprehensive Cancer Network (NCCN) suggest that adjuvant chemotherapy with trastuzumab should be considered in patients with pT1abN0 (or N1mi) HER2+ tumors [24]. van Ramshorst et al., using data from the Netherlands Cancer Registry, reported that treatment benefits in patients given adjuvant chemotherapy and/or trastuzumab were similar in all three early-stage breast tumor groups, with 8-year BCSS estimates of 100 versus (vs.) 95% in T1a (HR 0.05; 95% CI 0–8.81 ×10^3^; *p *= 0.62), 99 vs. 94% in T1b (HR 0.25; 95% CI 0.03–1.88; *p *= 0.18), and 96 vs. 90% in T1c tumors (HR 0.34; 95% CI 0.22–0.52; *p *< 0.001), and OS estimates of 100 vs. 85% in T1a (HR 0.05; 95% CI 0–1.99 × 10^2^; *p* = 0.47), 99 vs. 89% in T1b (HR 0.14; 95% CI 0.02–0.99; *p *= 0.05), and 94 vs. 80% in T1c tumors (HR 0.23; 95% CI 0.16–0.33; *p *< 0.001) [25]. Tolaney et al. published that in the APT phase II clinical trial, 12 weeks of paclitaxel + trastuzumab followed by 1 year of trastuzumab monotherapy in node-negative HER2+ tumors less than 3 cm (with 18.9% and 30.5% of patients having T1mi/a and T1b tumors, respectively), demonstrated excellent survival outcomes (DFS 93.3% and OS 95.0% at 7 years) [26, 27]. Furthermore, they recently described a low incidence of grade 3 to 4 left ventricular systolic dysfunction (0.5%) and left ventricular ejection fraction decline (3.2%) during treatment with 4-year median follow-up [28]. However, long-term toxic effects should be taken into consideration in the final decision process. In light of results of our study, our opinion is that treatment of patients with pT1ab HER2+ tumors with trastuzumab-based adjuvant therapy should be discussed.

Our study showed that younger patients (< 35 years) even with pT1b tumors had a poor prognosis, as shown in Table 2 (DFS: HR 3.18; 95% CI 1.08–9.34; *p* = 0.036). Therefore, we may consider treating younger patients with pT1b tumors because there was no difference regarding treatment ratios among the subgroups defined according to age at diagnosis except for the > 70 years subgroup (Supplementary Fig. 3B). Moreover, elderly patients (> 70 years) with pT1c tumors had significantly poorer prognoses (DFS: HR 1.63; 95% CI 1.06–2.49; *P *= 0.026, and OS: HR 2.49; 95% CI 1.37–4.51; *p *= 0.003) and, therefore providing treatment to elderly patients is expected to have a significant beneficial survival effect. Sawaki et al. reported in a randomized controlled trial (N-SAS BC07/RESPECT) that trastuzumab monotherapy could be an option as an adjuvant therapy for elderly (70–80 years) HER2+ breast cancer patients in light of less toxicity and a better quality of life compared with chemotherapy plus trastuzumab [29]. Of the 266 patients, 49% were T1b and T1c, and DFS at 3 years was 94.8% in the chemotherapy plus trastuzumab-treated group and 89.2% in the trastuzumab-treated group (HR 1.42; 95% CI, 0.68–2.95; *p *= 0.35). Based on our results, we suggest that the same treatment options should be discussed for all patients with pT1N0 HER2+ tumors, regardless of tumor size or age.

Our data showed that patients with pT1c tumors that were ER− had a poor prognosis (DFS: HR 2.01; 95% CI 1.27–3.16; *p *= 0.0027, BCSS: HR 3.93; 95% CI 1.41–11.0; *p *= 0.0089, and OS: HR 2.58; 95% CI 1.27–5.24, *p *= 0.0088). However, regarding pT1ab tumors, being ER- was not a predictive marker. In Japan, physicians tend to avoid adjuvant chemotherapy for patients with ER+/HER2+ tumors (Supplementary Fig. 3A). van Ramshorst et al. demonstrated that improved OS and BCSS outcomes were seen with systemic treatment in both hormone receptor-positive and -negative subgroups with a relative risk reduction in the same range, but that the absolute benefit was smaller in the hormone receptor-positive subgroup [25]. The difference can be explained by the use of adjuvant endocrine therapy for both hormone receptor-positive subgroups, regardless of chemotherapy and/or trastuzumab treatment. According to a meta-analysis of the randomized trial data by O’Sullivan et al., addition of trastuzumab to the chemotherapy improved DFS and OS in 4220 patients with early-stage HER2+ tumors, half of whom had none or one positive lymph node, and the proportional benefit was similar in both hormone receptor-positive and -negative diseases [30]. In the APT trial, the 3-year rate of recurrence-free survival (RFS) was 99.2% (95% CI 98.4–100), and there was no difference in subgroups defined according to tumor size (≤ 1 cm vs. > 1 cm) or hormone receptor status (positive vs. negative) [27]. Therefore, our viewpoint is that treatment with chemotherapy and/or trastuzumab for patients with pT1c tumors, particularly those that are ER−, is warranted.

The use of endocrine therapy combined with HER2-targeted therapy implies a type of dual blockade for patients with ER+/HER2+ tumors. Dackus et al. have recently published that aromatase inhibitors (AIs) (with or without ovarian ablation) were associated with better RFS and OS outcomes in ER+/HER2+ perimenopausal (aged 45–55 years) breast cancer patients, using data from the NCR [31]. According to multivariate Cox regression analysis, premenopausal women derived a significant survival benefit from AIs compared with tamoxifen (RFS: HR 0.47; 95% CI 0.25–0.91, *p *= 0.03 and OS: HR 0.37; 95% CI 0.18–0.79, *p *= 0.01). It is essential to use endocrine therapy combined with HER2-targeted therapy concurrently or sequentially. Moreover, an antibody–drug conjugate of trastuzumab and the toxic agent, trastuzumab-emtansine (T-DM1), is expected to replace trastuzumab-based treatment in various clinical situations. The phase II ATEMPT trial is ongoing, and patients with stage I HER2+ tumors are assigned to receive paclitaxel and trastuzumab followed by 1 year of trastuzumab or T-DM1 [32]. von Minckwitz et al. have recently published that the risk of recurrence of invasive breast cancer or death was 50% lower with adjuvant T-DM1 than with adjuvant continuation of trastuzumab alone for patients with HER2+ early breast cancer who had residual invasive disease after completion of neoadjuvant therapy [33]. Adverse events of grade 3 or higher were more common in the T-DM1 than in the trastuzumab group (25.7 vs. 15.4%). In light of the above, careful patient selection is most important when making decisions on a treatment regimen to avoid unnecessary toxicity.

There are several important limitations to this study. First, it consisted of retrospectively collected cases, including the possibility of selection and precluding the determination of causal relationships. However, Japanese BCR data cover more than 50% of breast cancer patients diagnosed in Japan. Therefore, we do not expect that selection would substantially affect our findings. Second, data on histological grade and Ki-67 labeling index were not available. There was the possibility of unmeasured confounding regarding this point. Finally, our data were not centrally reassessed on ER, PR, or HER2 status. However, the strength of our study is that it draws from more than 50,000 patients treated by qualified doctors and institutions in a ‘real-world’ setting and that it has internal and external validity. Our goal is to be able to identify patients with early-stage HER2+ tumors who require systemic treatment including HER2-targeted agents.

## Conclusions

In conclusion, our findings demonstrate that systemic treatment and/or trastuzumab may not be necessary in the clinical management of pT1a HER2+ tumors. However, these treatments may be beneficial for pT1b tumors compared with no treatment. These results are compatible with various guidelines such as of those of the NCCN. Additional research using clinical registry data may be essential for verifying guideline treatments for this subgroup of breast cancer patients.

## Electronic supplementary material

Below is the link to the electronic supplementary material.

Supplementary material 1 (PDF 558 kb)

## Abbreviations

HER2: Human epidermal growth factor receptor 2
BCR: Breast Cancer Registry
NCD: National Clinical Database
JBCS: Japanese Breast Cancer Society
IHC: Immunohistochemistry
ER: Estrogen receptor
PR: Progesterone receptor
DFS: Disease-free survival
BCSS: Breast cancer-specific survival
OS: Overall survival
HR: Hazard ratio
CI: Confidence interval
NCCN: National comprehensive cancer network
vs.: Versus
RFS: Recurrence-free survival
AIs: Aromatase inhibitors
T-DM1: Trastuzumab-emtansine
