# Supplementary material for: A population-based recurrence risk management study of patients with pT1 node-negative HER2+ breast cancer: a National Clinical Database study
Source: Breast Cancer Res Treat. 2019 Aug 26;178(3):647–56. doi: 10.1007/s10549-019-05413-7 (PMC6817748; doi:10.1007/s10549-019-05413-7)

Supplementary Fig 1. Overall survival curves according to HER2 positivity in cohort 1

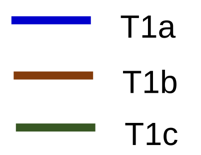

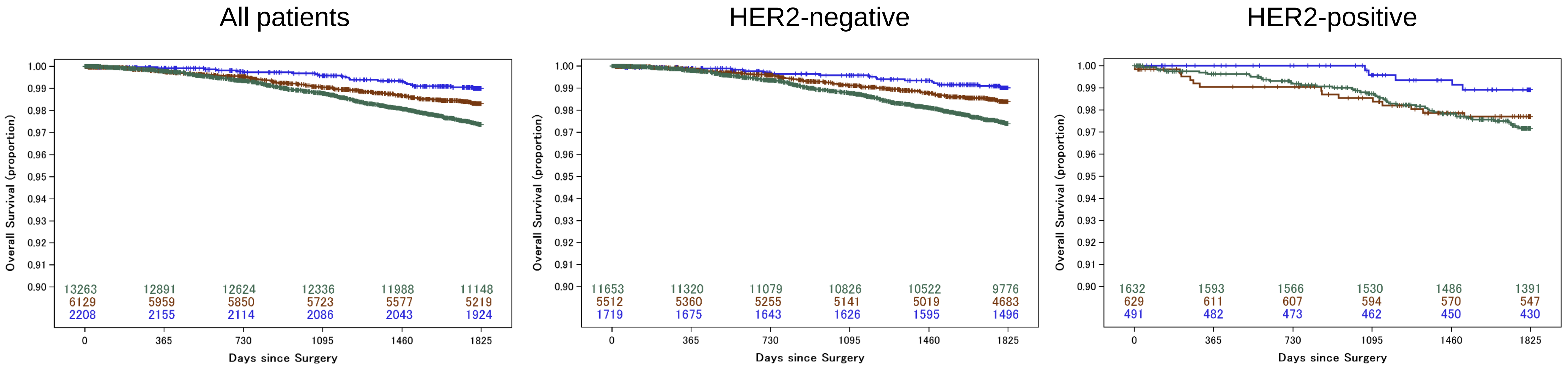

Supplementary Fig 2. Overall survival curves according to subtype classification in cohort 1

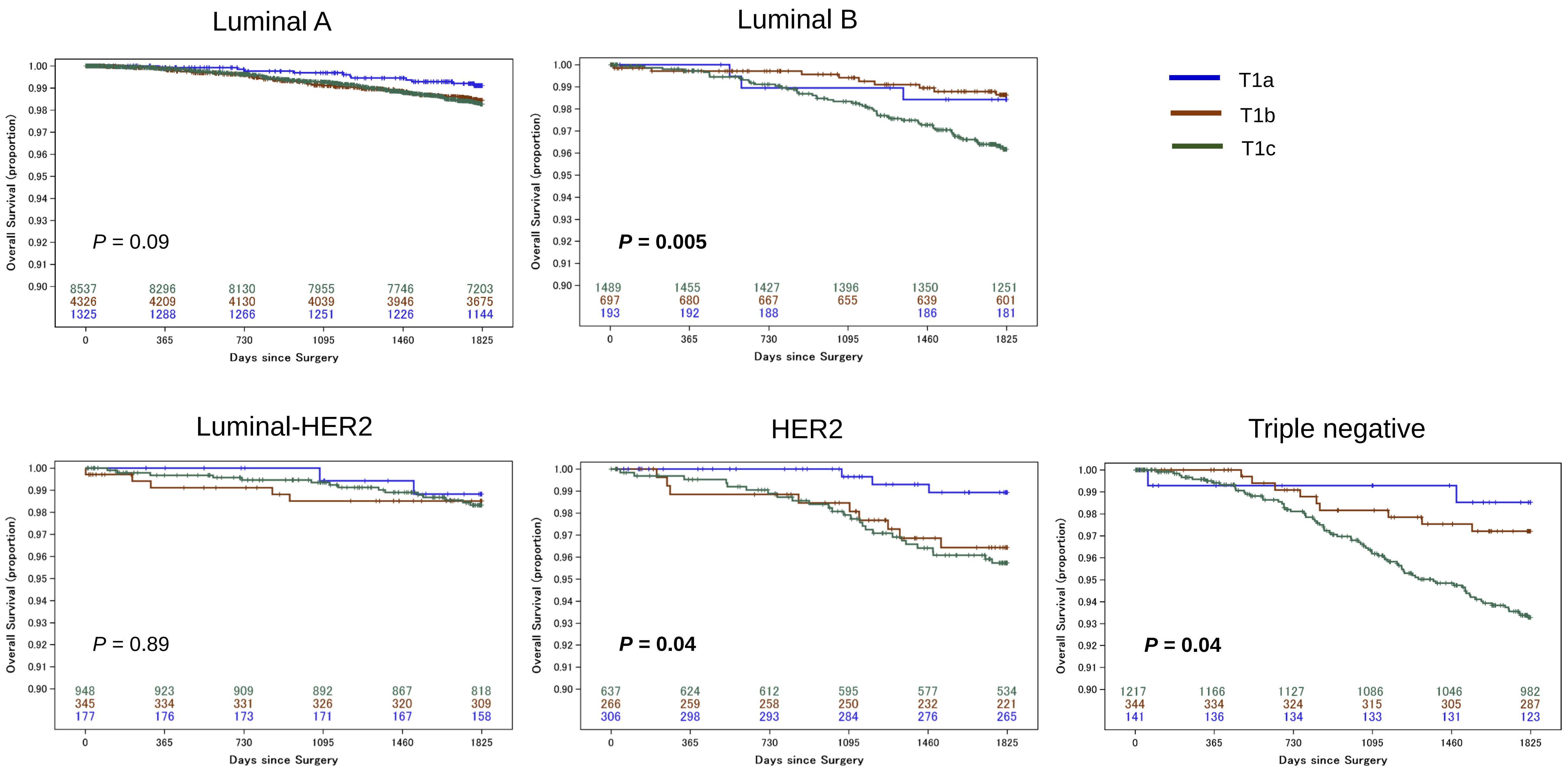

Supplementary Fig 3. Treatment ratio according to tumor size and ER status (A), and according to age at diagnosis and tumor size (B).

A.

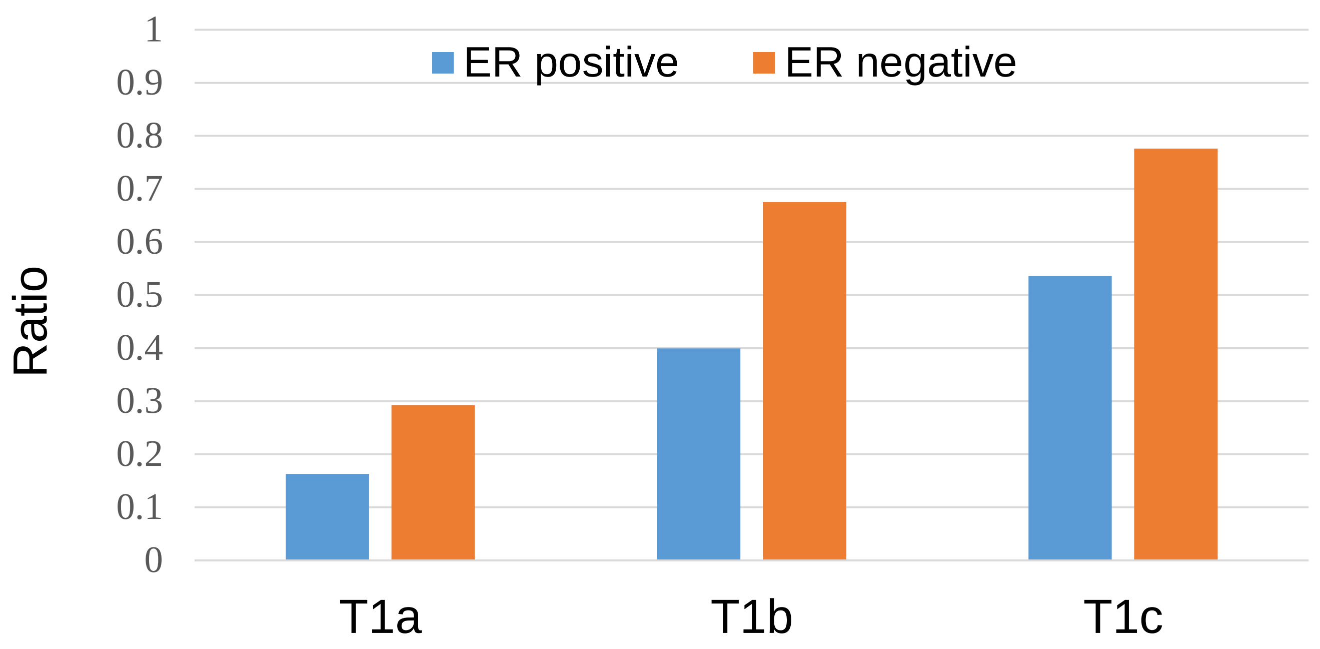

B.

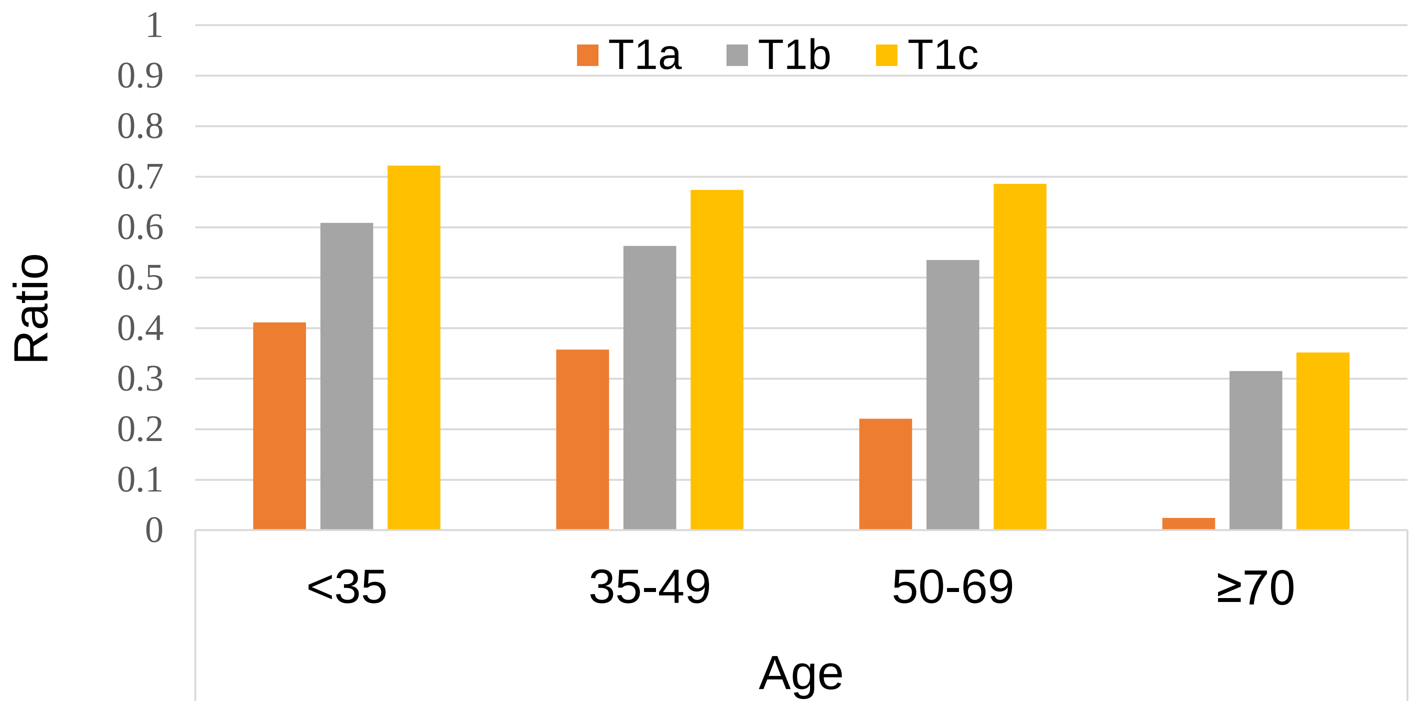

Supplement: Supplementary file 1 — Supplementary material 1 (PDF 558 kb) [file 10549_2019_5413_MOESM1_ESM.pdf]
